# Efficacy and safety of vitamin D supplementation in hospitalized COVID-19 pediatric patients: A randomized controlled trial

**DOI:** 10.3389/fped.2022.943529

**Published:** 2022-07-25

**Authors:** Jessie Zurita-Cruz, Jeffry Fonseca-Tenorio, Miguel Villasís-Keever, Mardia López-Alarcón, Israel Parra-Ortega, Briceida López-Martínez, Guadalupe Miranda-Novales

**Affiliations:** ^1^Faculty of Medicine, National Autonomous University of Mexico, Pediatric Hospital Federico Gómez, Mexico City, Mexico; ^2^Infectious Diseases Department, Pediatric Hospital National Medical Center, XXI Century, Mexican Institute of Social Security, Mexico City, Mexico; ^3^Analysis and Synthesis of the Evidence Research Unit, National Medical Center XXI Century, Mexican Institute of Social Security, Mexico City, Mexico; ^4^Medical Nutrition Research Unit, Pediatric Hospital National Medical Center, XXI Century, Mexican Institute of Social Security, Mexico City, Mexico; ^5^Clinical Laboratory Department, Hospital Infantil de México Federico Gómez, Mexico City, Mexico; ^6^Auxiliary Diagnostic Services, Hospital Infantil de México Federico Gómez, Ministry of Health, Mexico City, Mexico

**Keywords:** COVID-19, SARS-CoV-2, vitamin D, children, Pediatrics, mortality, Latin America

## Abstract

**Background:**

Some studies suggested that adequate levels of vitamin D (VD) decrease the risk of severe COVID-19. Information about the effectiveness of VD supplementation in children is scarce.

**Objective:**

To assess the efficacy and safety of VD supplementation compared to the standard of care in hospitalized children with COVID-19.

**Patients and methods:**

An open-label randomized controlled single-blind clinical trial was carried out. We included patients from 1 month to 17 years, with moderate COVID-19, who required hospitalization and supplemental oxygen. They were randomized into two groups: the VD group, which received doses of 1,000 (children < 1 year) or 2,000 IU/day (from 1 to 17 years) and the group without VD (control). The outcome variables were the progression of oxygen requirement, the development of complications, and death.

**Statistical analysis:**

For comparison between groups, we used the chi-squared test or Fisher's exact test and the Mann–Whitney *U* test. Absolute risk reduction (ARR) and the number needed to treat (NNT) were calculated. *p* ≤ 0.05 was considered statistically significant.

**Results:**

From 24 March 2020 to 31 March 2021, 87 patients were eligible to participate in the trial; 45 patients were randomized: 20 to the VD group and 25 to the control group. There was no difference in general characteristics at baseline, including serum VD levels (median 13.8 ng/ml in the VD group and 11.4 ng/ml in the control group).

**Outcomes:**

2/20 (10%) in the VD group vs. 9/25 (36%) in the control group progressed to a superior ventilation modality (*p* = 0.10); one patient in the VD group died (5%) compared to 6 (24%) patients in the control group (*p* = 0.23). ARR was 26% (95% CI 8.8 to 60.2%) and NNT was 3 (2 to 11) for progression and ARR was 19% (95% CI −3.9 to 42.8%) and NNT was 6 (2 to 26) for death. None of the patients receiving VD had adverse effects. The trial was stopped for ethical reasons; since after receiving the results of the basal VD values, none of the patients had normal levels.

**Conclusion:**

In this trial, VD supplementation in pediatric patients seems to decrease the risk of COVID-19 progression and death. More studies are needed to confirm these findings.

**Clinical Trial Registration:**

This protocol was registered on ClinicalTrials.gov with the registration number NCT04502667.

## Introduction

To date, there is no approved effective treatment for COVID-19 in hospitalized pediatric patients. Most of the available guidelines and consensus recommend supportive measures, supplemental oxygen, and consider the use of corticosteroids in some patients with respiratory compromise (1, 2). Although multiple treatments have been evaluated since the beginning of the COVID-19 pandemic: chloroquine or hydroxychloroquine, azithromycin, ivermectin, lopinavir/ritonavir, convalescent plasma, intravenous immunoglobulin, and interleukin (IL)-6 inhibitors, none of them have proven efficacy and safety in the pediatric population. However, despite the evidence in pediatric patients being insignificant and of low quality (3, 4), some guidelines recommend the use of remdesivir in critically ill patients (2, 5).

Most cases of COVID-19 in pediatric patients follow a mild course and predominate in school-age children. The benign behavior of the disease has not been fully explained. It has been proposed that repeated exposure in pediatric age to viral infections contributes to the strengthening of the immune system and a better response to severe acute respiratory syndrome coronavirus 2 (SARS-CoV-2). Also, angiotensin-converting enzyme 2 (ACE2) is a receptor through which SARS-CoV-2 enters the host cells; there is increasing evidence that a high level of ACE-2 expression is rather beneficial than harmful in lung injury. ACE-2 expression is high in infants, reaching a plateau in adolescence, and decrease during adulthood (6, 7). Although many of the cases in children are mild (8), in an analysis carried out with data from patients in Mexico City who require hospital treatment, the reported lethality was high, especially in the presence of comorbidities (9).

Vitamin D (VD) has a role in the pathogenesis of infectious diseases (10). In addition to acting directly against microorganisms, both the monocytes, innate antigen-presenting cells (APCs), and dendritic cells (DCs) are important targets for the immunomodulatory effects of VD. APCs are responsible for initiating the adaptive immune response, as they present antigens for T and B cells and are able to modulate them using immunogenic signals, such as cytokines and the expression of co-stimulatory molecules (11, 12). Different studies have shown that calcitriol and its analogs can alter the function and morphology of DCs to induce a more tolerogenic immature state (13, 14). Calcitriol has also been described as inhibiting T-cell cytokines, such as IL-2 and IL-17, and producing similar receptors on monocytes (15). In addition, in respiratory infections, VD increases the synthesis of the antimicrobial peptide cathelicidin in the respiratory epithelium (16), which has been shown to reduce disease severity and influenza virus replication *in vitro* (17).

Although several randomized controlled trials (RCTs) have attempted to assess the effect of VD supplementation on the prevention of respiratory tract infections (18–21), conclusions have been hampered by a small sample size, short trial duration, and lack of laboratory-confirmed results. In children, a systematic review of seven clinical trials found no association between VD supplementation and the prevention of acute respiratory infections in childhood (22).

Several observational studies have not only shown that hospitalized patients with COVID-19 have low levels of 25-hydroxyvitamin D3, but also that there is an inverse relationship between levels and disease severity (23–26). Therefore, RCTs have been carried out to evaluate the efficacy of VD administration; so far, these studies have been carried out in adult patients with inconsistent results (27–29).

The objective of this study was to evaluate the efficacy and safety of the VD supplementation (doses of 1,000 IU/day in children < 1 year and 2,000 IU/day in children 1 to 17 years old) in comparison with the standard of care in hospitalized children with COVID-19.

## Patients and methods

An open-label randomized controlled single-blind clinical trial was carried out in the Pediatric Hospital, National Medical Center, XXI Century. This hospital is a tertiary care center of the Mexican Social Security Institute (IMSS) and during the COVID-19 pandemic, it was one of the three pediatric hospitals in Mexico City designated to provide care for patients with COVID-19. Before the conversion, the hospital had 184 beds and two intensive care units (neonatal and pediatric). With the emergency, a special in-patient unit with 6 beds and one area with 40 beds were enabled to receive patients with SARS-CoV-2 infection. Any patient with acute respiratory disease or suspected SARS-CoV-2 infection was evaluated at the emergency department, regardless of the health insurance status.

This protocol was approved by the Institutional Review Board (R-2020-3603-20) and registered on ClinicalTrials.gov with the registration number NCT04502667. This trial was conducted in agreement with the ethical principles in the Declaration of Helsinki. All the parents or legal guardians of the participants provided written informed consent. For children over 8 years of age, assent was also requested. This study follows the Consolidated Standards of Reporting Trials (CONSORT) guideline.

Patients were enrolled from 24 March 2020 to 31 March 2021. Inclusion criteria were as follows: patients younger than 18 years old, with confirmed SARS-CoV-2 infection by real-time reverse transcription-PCR (rRT-PCR), requiring hospitalization and supplemental oxygen. Exclusion criteria were as follows: VD supplementation in the previous 7 days or contraindications for enteral feeding, while those patients that did not receive at least 7 days of VD supplementation were not included in the final analysis.

### Intervention

Patients were randomly assigned in a 1:1 allocation fashion to the VD group (VDG) or to the control group (CG) that did not receive VD. The VD group received doses of 1,000 IU/day for children younger than 1 year (One Drop^®^ Teriana Labs SA de CV, Mexico) and 2,000 IU/day for children 1 to 17 years (Histofil^®^, Medix Labs SA de CV, Mexico) during hospitalization for a minimum of 7 days and a maximum of 14 days. Randomization was conducted using web software (Research Randomizer: https://www.randomizer.org/) by a researcher who had no contact with participants. One of the researchers safeguarded the assignment sequence. Each time a new patient was included he oversaw delivering the sealed envelope to the attending physicians to assign the maneuver that corresponded.

Disease severity was classified as follows: (a) mild, in case of the presence of fever and/or asthenia and/or symptoms compatible with the upper respiratory infection without respiratory distress and/or instrumental evidence of pneumonia; (b) moderate, in the presence of respiratory distress and/or reduced nutrition and hydration and/or instrumental evidence of pneumonia; (c) severe, in the presence of severe respiratory distress and/or desaturation [oxygen saturation (SpO_2_) < 92% in ambient air] and/or intermittent cyanosis or apnea and/or systemic symptoms, such as lethargy, dehydration, convulsions, or suspected sepsis; and (d) critical, if acute respiratory distress syndrome (ARDS), multiorgan failure (MOF), septic shock, or coma occurs (30).

### Laboratory biomarkers

C-reactive protein (CRP), D-dimer (DD), and fibrinogen were compared at admission and 7th day of hospitalization. The serum concentration of 25-hydroxyvitamin D3 was determined with the method described in a study by Van Den Ouweland et al. (27) using the Waters ACQUITY UPLC Class coupled to a Xevo TQD (Waters, Milford, Massachusetts, USA), with an APCI Ion SABER II probe. A solid-phase extraction cartridge (Strata C18-E; 55 μm, 70 A, Phenomenex, California, USA) was used. Chromatographic analyses were performed with a C18 column at 50°C (Kinetex 1.7 μm XB-C18 100 A LC Column 50 mm × 2.1 mm, Phenomenex, California, USA). Data acquisition and analyses were performed using the Masslynx version 4.1 software (Waters). The 25-hydroxyvitamin D3 was analyzed in duplicate in a subsample of serum specimens and the coefficient of variation was 5.16%. It was considered VD deficiency when serum level was < 20 ng/ml, insufficiency with a level between 20 and 29.99 ng/ml, and normal with a level ≥30 ng/ml (31).

The laboratory diagnosis of SARS-CoV-2 infection by rRT-PCR was established in accordance with the Standardized Guidelines for Epidemiological Surveillance by a certified COVID-19 laboratory according to the standardized protocol (32).

The outcome variables were the progression of oxygen requirement, the development of complications, and death. Adverse effects of VD were also monitored: nausea, anorexia, vomiting, abdominal pain, diarrhea, headache, dizziness, constipation, muscle weakness, and rash. In case of presenting any of the symptoms attributed to the administration of VD, supplementation was discontinued immediately. Patients were followed from hospital admission to discharge or death during their hospitalization period.

### Statistical analysis

The sample size was calculated considering that patients with COVID-19 infection who do not receive doses of VD will have a higher relative risk (RR) of 1.67 of progressing to severe COVID-19 or dying compared to the control group. A total of 59 patients per group were estimated.

Statistical analyses were performed with SPSS version 24.0 (IBM Incorporation). Qualitative variables are expressed as proportions and quantitative variables are expressed as medians and interquartile ranges (IQRs), since they did not show a normal distribution. For the comparison between groups, we used the chi-squared test or Fisher's exact test and the Mann–Whitney *U* test. A multiple regression model was performed to determine the effect of VD supplementation on changes in laboratory markers. Absolute risk reduction (ARR) and the number needed to treat (NNT) were also calculated by intention to treat analysis. *p* < 0.05 was considered statistically significant.

## Results

**Figure 1:**
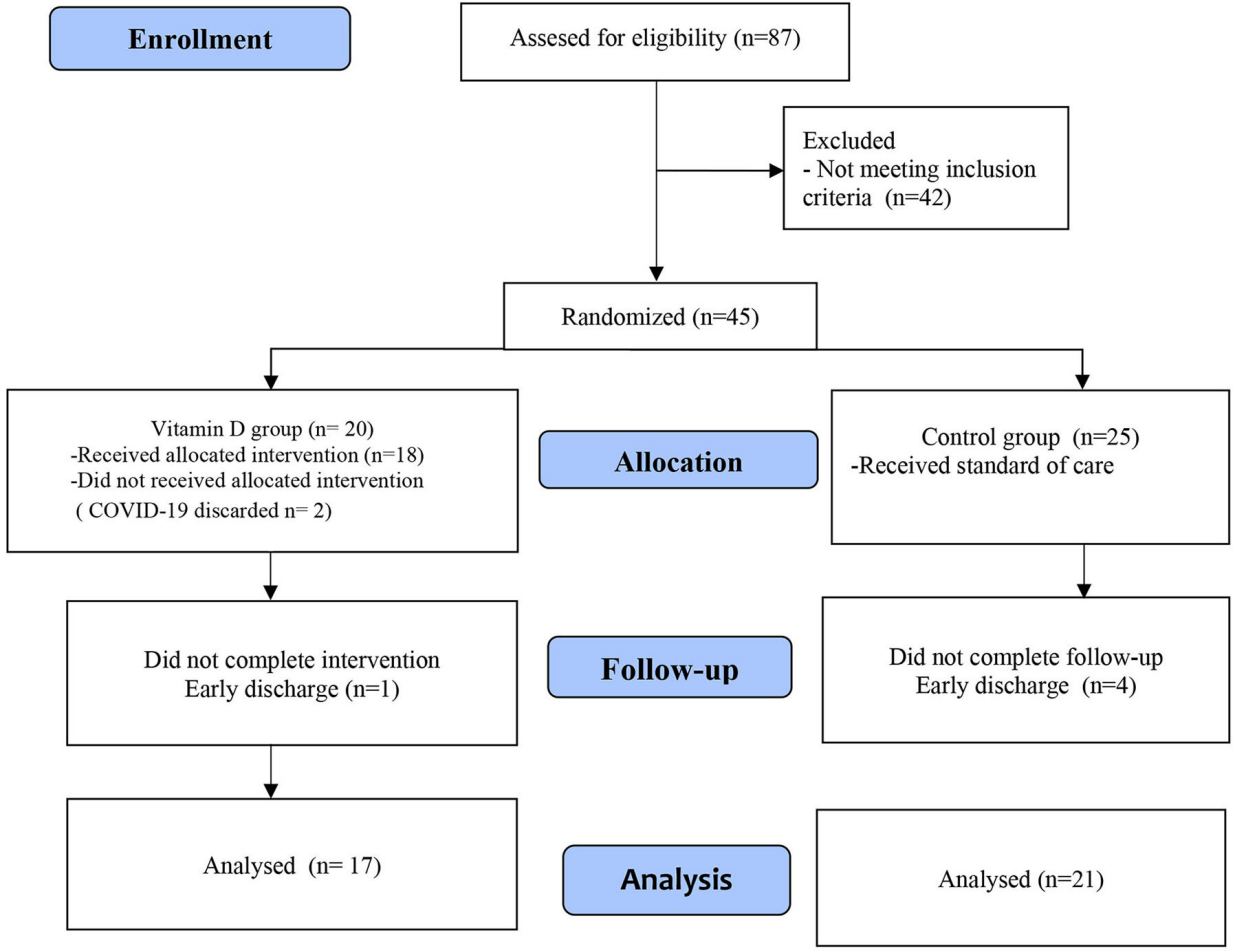
CONSORT flow diagram.

From 24 March 2020 to 31 March 2021, 697 patients who met the operational definition of a suspected case of COVID-19 were evaluated. There was a total of 260 patients with a positive rRT-PCR test, 173 patients had mild disease and were treated as outpatients (66.6%), and 87 patients were hospitalized (33.4%). Of the 87 patients who were hospitalized, the median age was 12 years (12 months to 17 years), 64.4% were male, and 81% had some comorbidity; of the eligible patients, 42 patients were excluded for not meeting the inclusion criteria (not requiring supplemental oxygen). The remaining 45 patients were allocated to one of the two groups, 20 to the VDG and 25 to the CG. In the VDG, 2/20 patients were excluded because of a negative rRT-PCR test. At follow-up, one patient received <7 days of VD supplement and four CG patients did not complete the intervention, as they were discharged before the 7^th^ day of hospitalization. Thus, 17 patients from the VDG and 21 patients from the CG were included in the final analysis (Figure 1).

Female sex predominated in participants; the median age for those in the VDG was 10.6 years, while for those in the CG was 13.9 years. It is noteworthy that, in both the groups, nearly half were obese. Of the patients who received VD supplementation, 45% (*n* = 9) had comorbidity in addition to obesity, the most frequent were cancer and gastrointestinal diseases (five and four patients, respectively), while for the control group, 72% *(n* = 18) had comorbidity, also the most frequent was cancer (six patients) followed by respiratory chronic disease (four patients). As shown in Table 1, except for the severity of COVID-19, there was no difference between the groups in the characteristics of the participants. In the CG (56%), there was a higher proportion of severe and critical patients than in the VDG (15%) (*p* = 0.03).

**Table 1:** Baseline characteristics of patients allocated to the intervention.

**Participants**	***N*** = **45**				***p*** **value**
**Group**	**Vitamin D supplementation** ***N*** = **20**		**Control** ***N*** = **25**		
Age in years	Median 10.66/ IQR 10.25 (4.41–14.62)		Median 13.95/ IQR 7.52 (7.35–14.87)		0.56
Sex	Male 9 (45%)	Female 11 (55%)	Male 9 (36%)	Female 16 (64%)	0.77
Body Mass Index (BMI)	Median 24/ IQR 10.76 (18.75–29.51)		Median 24.32/ IQR 11.73 (18.5–30.23)		0.82
Nutritional status					0.40
Obesity	10 (50%)		10 (40%)		
Overweight	5 (25%)		5 (20%)		
Normal	3 (15%)		7 (28%)		
Undernutrition	2 (10%)		3 (12%)		
Hospital stay (days)	Median 9 / IQR 7.5 (7–14.5)		Median 11 / IQR 9.5 (6–15.5)		0.79
Previously healthy	10 (50%)		7 (28%)		
Chronic disease or underlying condition	9 (45%)		18 (72%)		0.08
Cancer	5 (25%)		6 (24%)		
Congenital hyperinsulinism	0 (0%)		1 (4%)		
Diabetes mellitus (cetoacidosis)	0 (0%)		1 (4%)		
Gastrointestinal disorders (gastroesophageal reflux)	4 (20%)		2 (8%)		
Pulmonary diseases	0 (0%)		4 (16%)		
Neurological diseases	1 (5%)		1 (4%)		
Cardiovascular diseases	0 (0%)		2 (8%)		
Down Syndrome	0 (0%)		1 (4%)		
Disease severity classification					0.03
Moderate	17 (85%)		11 (44%)		
Severe	3 (15%)		10 (40%)		
Critical	0 (0%)		4 (16%)		

IQR, Interquartile range.

The main clinical picture was characterized by cough and respiratory distress, as well as evidence of pneumonia on physical examination. The 45 patients assigned to the intervention had an X-ray upon admission; the main radiological findings were peripheral reticular interstitial pattern (28.8%), followed by bilateral micromacronodular infiltrates, bilateral peripheral small opacities, reticular pattern (17.7% each), and glass-ground opacities (3.6%). Among other findings found, air bronchogram (11.1%), cardiomegaly (6.6%), increased pulmonary flow (4.4%), and pleural effusion (2.2%) were observed, although these were related to underlying pathologies. Up to 11.1% (5 patients) had a normal X-ray. Table 2 shows the characteristics of each group separately. The number of alterations is higher than the total number of patients because several patients had more than one finding.

**Table 2:** Radiologic findings in study participants.

	**Vitamin D supplementation** **group** ***N*** = **20**	**Control group** ***N*** = **25**
Normal	0 (0%)	5 (20%)
Peripheral reticular interstitial pattern	7 (35%)	6 (24%)
Bilateral peripheral small opacities (nodules)	2 (10%)	6 (24%)
Bilateral micro-macronodular infiltrates	4 (20%)	4 (16%)
Reticular pattern	5 (25%)	3 (12%)
Glass-ground opacities	1 (5%)	1 (4%)
Other findings		
- Air bronchogram - Cardiomegaly - Increased pulmonary flow - Pleural effusion	2 (10%) 1 (5%) 0 (0%) 1 (5%)	3 (12%) 2 (8%) 2 (8%) 0 (0%)

Vitamin D levels in the baseline sample showed VD deficiency in the 45 patients, with a median of 11.7 ng/ml (IQR 9.4–17.22 ng/ml). Only four patients had an insufficient level. None of the patients had a normal level. By group, the VDG had a median level of 13.8 ng/ml (IQR 10.75–18.35 ng/ml) and the control group had a median level of 11.4 ng/ml (IQR 8.7–13.1 ng/ml).

This represented an ethical challenge; in agreement with the investigators and the ethics committee, it was decided to stop the trial and supplement VD to every patient with COVID-19 admitted to the hospital.

### Management and outcomes

As shown in Table 3, support treatment was somehow similar for both the groups. Patients with severe and critical diseases required assisted mechanical ventilation and received intensive care, which occurred more often in the control group (56 vs. 15%). Corticosteroids, an antibiotic use, were similar between groups. Oseltamivir was prescribed to 5 patients.

**Table 3:** Supportive care and antibiotic use in trial participants.

	**Vitamin D supplementation** **group** ***N*** = **20**	**Control group** ***N*** = **25**
Intensive care	3 (15%)	14 (56%)
Corticosteroids	10 (50%)	14 (56%)
Antibiotics	10 (50%)	12 (48%)
Diagnosis prescription		
- At admission for suspected bacterial pneumonia - Before hospitalization - Healthcare associated infection - Febrile neutropenia episode	4 2 4 0	3 0 6 3
Type of antibiotic		
- Penicillins - Cephalosporins - Beta-lactam/beta-lactamase inhibitor - Glycopeptide - Aminoglycoside	1 8 2 2 1	1 4 9 2 2
Antibiotics per patient Oseltamivir	1.4 3 (15%)	1.5 2 (8%)

Of the patients in the VD group, only two patients progressed to a superior ventilation modality compared to nine patients in the control group. This resulted in an absolute risk reduction (ARR) of 26% (95% CI 8.8–60.2%) and a number needed to treat (NNT) of 3 (2 to 11).

For mortality, in the VDG group, there was one death (related to COVID-19) compared to six deaths in the CG; of these six deaths, two deaths were from patients classified as critically ill. Four of the deaths were related to COVID-19 and two deaths were related to the underlying condition. The ARR was 19% (95% CI −3.9 to 42.8%) and NNT was 6 (2 to 26).

In patients that progressed to a superior ventilator modality, malnutrition (undernutrition and obesity) was higher than in children with a normal nutritional status (28.5 vs. 10%), although the difference was not statistically significant (*p* = 0.22). Of the seven patients who died, only one patient had a normal nutritional status.

None of the patients who received vitamin D had medication-related adverse effects.

Days of hospital stay for the VDG were lower (median 9 days) compared to those in the group with the standard of care treatment (median 11 days).

### Laboratory markers

**Figure 2:**
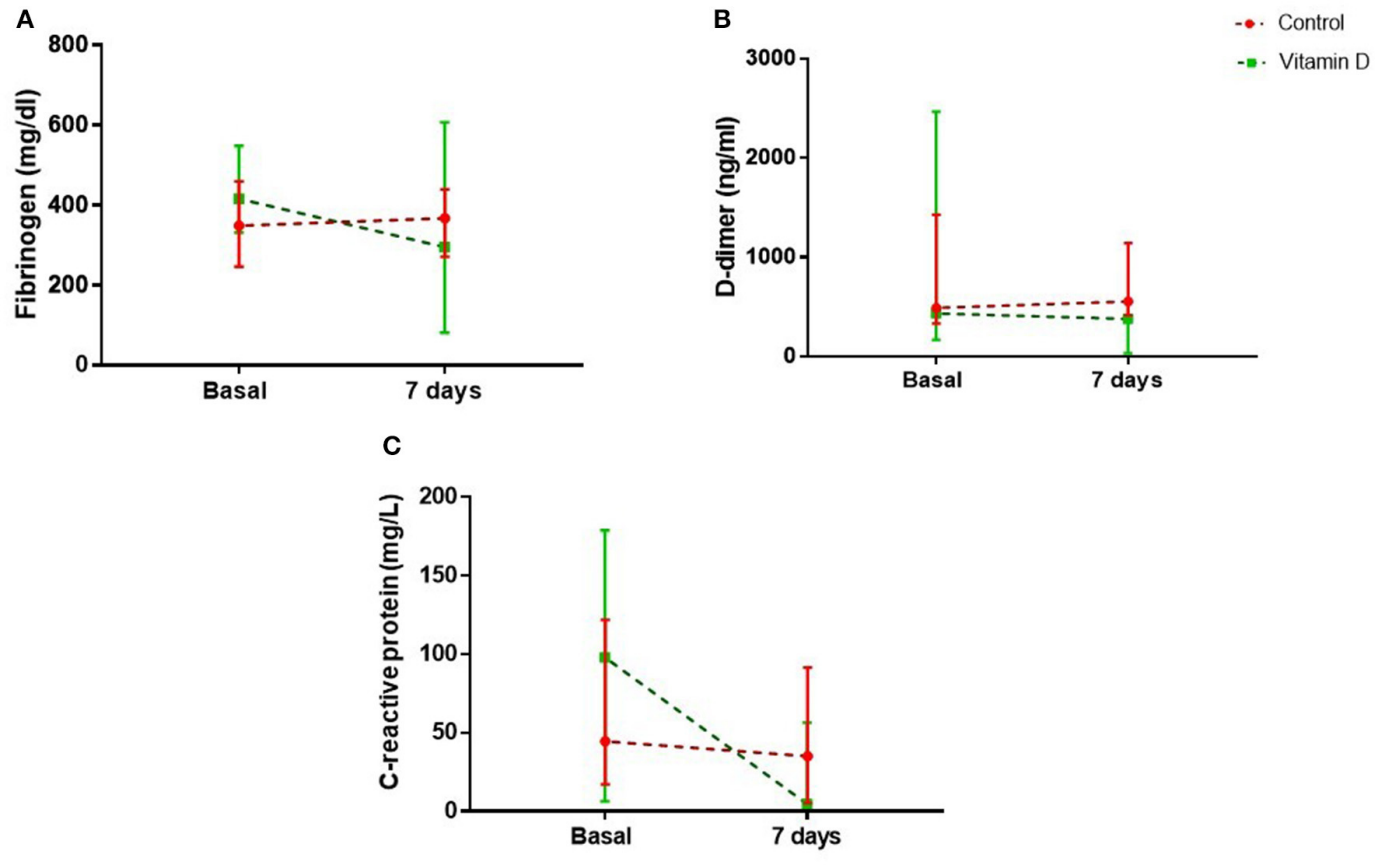
Comparison of basal and seventh day serum concentrations of fibrinogen **(a)**, D-dimer **(b)**, and C-reactive protein **(c)**, and in the control and vitamin D supplementation groups.

It was observed that in the VDG, there was a decrease in basal CRP value (97.9 vs. 3.0 mg/l, *p* = 0.007), without modification in the control group on 7^th^ day (44.3 vs. 34.9 mg/l, *p* = 0.683). Comparisons are difficult as not all the patients had basal and follow-up results. For patients in the VD group, CRP value at 24 h had a notable rise, with a median of 143.7 mg/l (minimum 76 mg/l and maximum 243 mg/l). There was no difference in DD and fibrinogen. In the linear regression model, it was identified that VD decreased the serum concentrations of CRP [coefficient −71.9 (95% CI −139.0 to −4.8), *p* = 0.037] (Table 4, Figure 2).

**Table 4:** Laboratory biomarkers comparison in the control group and the vitamin D supplementation group at admission and 7^th^ day follow-up.

	**Control group**			**Vitamin D group**			**Deltas**		
	**Basal**	**7 days**		**Basal**	**7 days**		**Control**	**Vitamin D**	
Biomarker	Median (Confidence interval 95%)		*p*	Median (Confidence interval 95%)		*p*	Median (Confidence interval 95%)		*p*
C-reactive protein mg/L	44.3 (37.4–109.2) [*n* = 20]	34.9 (20.6–124.1) [*n* = 15]	0.683	97.9 (37.3–173.7) [*n* = 14]	3.0 (1.5–43.6) [*n* = 11]	0.007	−5.8 (−51.5 to 48.4) [*n* = 14]	−80.0 (−108.3 to −33.3) [*n* = 11]	0.027
Fibrinogen mg/dl	350.5 (271.1–405.6) [*n* = 16]	362.5 (194.3–776.0) [*n* = 12]	0.678	415.5 (334.4–527.0) [*n* = 12]	330.0 (218.8–421.6) [*n* = 13]	0.136	−26.0 (−128.5 to 58.9) [*n* = 9]	−138.0 (−245.2 to 39.0) [*n* = 9]	0.233
D-dimer ng/ml	488.5 (380.1–1,571.2) [*n* = 16]	553.0 (65.4–2,034.1) [*n* = 9]	0.753	431.5 (204.0–2,159.9) [*n* = 14]	376.5 (214.6–491.4) [*n* = 8]	0.500	161.0 (−1,039.0 to 1,878.6) [*n* = 6]	−29.0 (−2,620.0 to 1,252.1) [*n* = 5]	0.583

## Discussion

Most studies report that previously healthy pediatric population with SARS-CoV-2 infection has a favorable and uncomplicated evolution, but patients with chronic diseases or underlying conditions are at risk of presenting with severe COVID-19 and dying as consequence. So, it is necessary to recognize and evaluate therapeutic measures in different scenarios and determine if some strategies used in other viral respiratory diseases are effective for COVID-19 in children.

This RCT is one of the first trials to evaluate the effectiveness of VD supplementation to improve the clinical conditions of children with COVID-19 requiring hospitalization. The results seem to show that there is some benefit from VD, since there were fewer patients who progressed in disease severity and died.

Ramírez-Sandoval et al. (33) found that in a cohort of 2,908 Mexican adult patients with COVID-19, only 19.6% showed VD deficiency (<12.5 ng/ml) and 36% had levels between 12.5 and 20 ng/ml, different from our study, where age alone appears to be a risk factor for vitamin D deficiency. Rustecka A et al. also found in 1,472 children from Poland that the proportion of children with VD deficiency increased after several months of the COVID-19 pandemic (34) similar to the study by Kang et al. (35) in Seoul, Korea. In contrast, Meoli M et al. compared VD levels with data previously obtained in 473 adolescents and young adults in Switzerland, in the period 2014–2016, with a new group of young people (298, aged 18–19 years) in the period from July to December 2020, and found no difference in VD insufficiency (36). Alpcan et al. (37) in Turkey found that the serum VD level was significantly lower in a group of 75 hospitalized COVID-19 pediatric patients compared to the control group of 80 healthy children (21.5 ± 10.0 vs. 28.0 ± 11.0 IU, *p* < 0.001).

The role of VD in immune defense in COVID-19 has been studied, with different mechanisms taking part, such as the formation of an antimicrobial peptide in the respiratory epithelium (cathelicidin), producing chemotaxis-activating β defensins, differentiation of monocytes in macrophages, as well as the activation of oxidative pathways in monocytes and macrophages, an important antiviral mechanism, modulates the expression of pattern recognition receptors, interrupting local inflammation and releasing chemokines, inhibits the secretion of IL-12, IL-23, IL-10, as well as the expression of major histocompatibility complex (MHC) class II molecules and stimulators, such as CCL4 and CCL19, decreasing the inflammatory response (38).

Vitamin D supplementation in adult patients has been evaluated in RCTs, both as a prevention of disease acquisition and as an adjuvant for the improvement of hospitalized patients. Karonova et al. (39) analyzed the potential effect of vitamin D supplementation in reducing SARS-CoV-2 infection morbidity and severity in healthcare workers, but neither vitamin D intake nor vitamin D deficiency/insufficiency was associated with a decrease in SARS-CoV-2 morbidity [odds ratio (OR) 2.27; 95% CI, 0.72 to 7.12]. Meanwhile, Murai et al. reported that the hospital length of stay of patients with COVID-19 was similar between those who received a high single VD dose compared to those who did not receive a high single VD dose (40). In contrast, Nogues X et al. found that weekly high-dose calcifediol decreased severity and mortality in patients with COVID-19 (41).

Available systematic reviews and meta-analyses have not reached definitive conclusions. In the meta-analysis published in a study by Oscanoa TJ et al. (42), which included 23 studies (*n* = 2,692 subjects, predominantly male adults), it was found that vitamin 25(OH)D deficiency was associated with an increased risk of severe COVID-19 (RR 2.00; 95% CI 1.47–2.71, 17 studies) and mortality (RR 2.45; 95% CI 1.24–4.84, 13 studies). In a later meta-analysis, including six RCTs (551 adult patients with COVID-19), the results suggest an overall beneficial effect of VD treatment when all the observations across all the RCTs were pooled as an overall effect size. The authors consider that the difference in the studies settings, timings, randomization, blinding, and data collection strategies could have influenced the outcomes (43).

Specifically, in the pediatric population, the systematic review conducted by Shah K et al. (44) aimed to estimate the prevalence of VD deficiency in pediatric patients with COVID-19 and the association between VD deficiency and disease severity, as well as the relationship between VD supplementation and improvement. Eight eligible studies (two reviews) were included in the review. Meta-analysis of the six studies showed a prevalence of VD deficiency of 45.91% (95% CI 25.14–67.45). The analysis of two studies showed that low VD levels increased the risk of severe disease (OR 5.5; 95% CI 1.56–19.51, *p* = 0.008). Due to limited and heterogeneously published literature, the effect of VD supplementation on COVID-19 infectivity and severity could not be explored, but authors consider that needs to be evaluated as a preventive measure in pediatric patients with COVID-19.

Probably, the benefit we observed in our population was due to the fact that most had VD deficiency and the impact of supplementation on severe COVID-19 was greater than reported in other studies where the percentage of the deficiency was lower.

One aspect to consider is the design of the study; although it was an open-label RCT without a placebo, the outcome measures were blindly evaluated. Daily monitoring of each patient was performed by the same two investigators, thereby achieving a standardized assessment. Other strengths of the present study are that all the patients but one in the group that received VD had complete therapeutic compliance and that there were no losses to follow-up.

Although there seems to be a beneficial effect of vitamin D supplementation, we must consider the study limitations. Undoubtedly, the main limitation was not having completed the sample size due to ethical reasons, which most likely conditioned that some variables were not distributed homogeneously between groups, such as the severity of COVID-19, comorbidities (e.g., pulmonary disease), as well as in the values of laboratory markers. Another limitation was that the serum levels of vitamin D were not quantified at the end of this study.

After more than 2 years of the COVID-19 pandemic and an immense amount of published literature on the role of vitamin D in COVID-19 severity, there is no definite answer to recommend vitamin D supplementation. Although several studies seem to support the supplementation with vitamin D in high-risk patients, the results are still inconsistent. To limit the risks for the participants, a different threshold value for serum vitamin D should be used. In this study, we used the values established by the Endocrine Society (45) to consider vitamin D deficiency (serum level < 20 ng/ml) or insufficiency (level between 20 and 29.99 ng/ml). If the criteria change using the values by the Food and Nutrition Board, children with insufficiency (level between 12 and 20 ng/ml) could participate in a new trial and only patients with vitamin D deficiency (<12 ng/ml) would be excluded. In the recent review by Briceno Noriega et al., several recommendations are made for future research (46).

The number of patients who did not have progression of the disease and the lower number of deaths in the group of patients who received VD is notorious. Therefore, the results do not allow to issue for a definitive recommendation, but in light of current evidence in pediatric hospitalized COVID-19 with limited specific treatment available, VD supplementation needs to be considered as an alternative to improve the outcome.

## Data availability statement

The raw data supporting the conclusions of this article will be made available by the authors, upon reasonable request.

## Ethics statement

The studies involving human participants were reviewed and approved by Comité Local de Ética en Investigación number 3603, authorization number R-2020-3603-20. Written informed consent to participate in this study was provided by the participants' legal guardian/next of kin.

## Author contributions

JZ-C, MV-K, ML-A, and GM-N: study design. JF-T, IP-O, BL-M, and GM-N: collection, analysis, and interpretation of data. JZ-C, GM-N, and MV-K: manuscript preparation and final approval. All the authors have contributed to the article and approved the submitted version of the manuscript.

## Conflict of interest

The authors declare that the research was conducted in the absence of any commercial or financial relationships that could be construed as a potential conflict of interest.

## Publisher's note

All claims expressed in this article are solely those of the authors and do not necessarily represent those of their affiliated organizations, or those of the publisher, the editors and the reviewers. Any product that may be evaluated in this article, or claim that may be made by its manufacturer, is not guaranteed or endorsed by the publisher.
